# Cancer Incidence in Kabul, Afghanistan: The First Report From the Population‐Based Cancer Registry

**DOI:** 10.1002/cam4.70844

**Published:** 2025-04-18

**Authors:** Maihan Abdullah, Nisar Niazi, Goodarz Danaei, Jesse Bump, Timothy R. Rebbeck, Ikram Hashimi, Sabira Niyazi, Marwa Sarwari, Fatemeh Ghasemi‐Kebria, Gholamreza Roshandel

**Affiliations:** ^1^ Ministry of Public Health Kabul Afghanistan; ^2^ Takemi Program in International Health, Harvard T.H. Chan School of Public Health Harvard University Boston Massachusetts USA; ^3^ Department of Global Health and Population, Department of Epidemiology Harvard T.H. Chan School of Public Health, Harvard University Boston Massachusetts USA; ^4^ Dana‐Farber Cancer Institute and Harvard TH Chan School of Public Health Boston Massachusetts USA; ^5^ Hayat Balkh Institute of Health Sciences Mazar‐e‐Sharif Balkh Afghanistan; ^6^ Golestan Research Center of Gastroenterology and Hepatology, Golestan University of Medical Sciences Gorgan Iran

**Keywords:** cancer in Afghanistan, cancer in Kabul, cancer incidence, cancer registry, common cancers in Kabul, population‐based

## Abstract

**Background:**

Establishing a population‐based cancer registry is crucial for understanding cancer incidence, identifying risk factors, and developing effective cancer control programs. The Kabul Cancer Registry (KCR), Afghanistan's first population‐based cancer registry, was established in 2018. The purpose of this study was to estimate the incidence of cancer in Kabul between 2018 and 2020.

**Methods:**

The KCR, adhering to International Agency for Research on Cancer (IARC) standards, actively collected data on new cancer cases from health facilities in Kabul between 2018 and 2020. We used CanReg5 software to calculate age‐standardized incidence rates (ASIRs) by cancer site in males and females, using the direct method with Segi's World Standard Population.

**Results:**

The KCR recorded 4498 new cancer cases among Kabul residents, with a male‐to‐female ratio of 0.82:1. The overall ASIR was 44.3 per 100,000 person‐years in males and 60.9 in females. The top five cancer sites in males were stomach (ASIR = 9.1), esophagus (ASIR = 5.5), colorectum (ASIR = 3.7), lymphoma (ASIR = 2.4), and liver (ASIR = 2.1). In females, the top five cancer sites were breast (ASIR = 14.9), esophagus (ASIR = 6.7), stomach (ASIR = 4.2), colorectum (ASIR = 3.8), and gallbladder (ASIR = 1.8). Childhood cancers (aged 0–14 years) represented 6.8% of all cancers, with leukemia accounting for 43.5% of the new cancer cases.

**Conclusions:**

The high incidence of breast, stomach, and esophagus cancers highlights the need for policymakers and healthcare providers to develop cancer control programs focused on primary prevention, early detection, and quality diagnosis and treatment. Additionally, this study underscores the importance of cancer registries and emphasizes the need to strengthen the KCR to improve data quality.

## Graphical abstract

This study examines cancer incidence in Kabul, Afghanistan, using data from the first population‐based cancer registry established in the region. It highlights the burden of cancer types most prevalent in the population, providing critical insights into the patterns and disparities compared to neighboring countries. The findings underscore the importance of localized data for developing effective cancer prevention, early detection, and control strategies in resource‐limited and conflict‐affected settings.

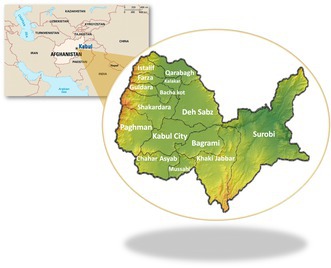

## 1 Introduction

Cancer poses a significant burden on society, with substantial societal and economic costs [1]. An estimated 19.96 million new cancer cases occurred in 2022 worldwide. It was also estimated that nearly half (49.2%) of all newly diagnosed cases and more than half (56.1%) of cancer deaths in 2022 occurred in Asia. The burden of cancer incidence and prevalence is growing rapidly and is one of the highest in developing countries, especially in Asia. It is projected that by 2050, the number of new cancer cases will reach approximately 35.0 million, representing a relative increase of more than 75% compared with that in 2022 [2].

In view of the increasing burden of noncommunicable diseases (NCDs), especially cancer, the Member States of the United Nations committed to the Sustainable Development Goals 2030 agenda to reduce premature deaths due to NCDs by one‐third [3]. The World Health Assembly endorsed an action plan to achieve that goal [4]. To monitor the action plan and assess the progress of national NCD strategies, including cancer strategies, the Member States agreed on 25 indicators. One of the indicators in the global framework document is the number of new cancer cases by type per 100,000 person‐years, age‐standardized [5].

The literature on the subject of cancer epidemiology in Afghanistan is limited, mainly because of the more than 40 years of conflict and lack of cancer control activities in the country. The first report on the frequency of cancer in Afghanistan described 895 tumors (550 malignant and 345 benign), which were collected between September 1965 and March 1968 (2.5 years) by the pathology‐based tumor registry at Kabul University. The most common cancers reported were those of the skin, lymphoid system, and soft tissues in males, whereas those of the breast, skin, and soft tissues were reported in females [6]. In recent years, some studies have described cancer patterns using data from merely individual health facilities located both in Kabul or outside [7, 8, 9, 10], as well as GLOBOCAN's estimates from the International Agency for Research on Cancer (IARC) [11].

While some of these studies provide a description of cancer in Afghanistan to some extent, only data from a population‐based cancer registry provide an accurate measure of the epidemiology of cancer [12]. Cancer registries are essential for monitoring not only new cancer cases but also the prevalence and mortality of cancer, the evaluation of national cancer control programs, public policy related to cancer control, and the allocation of resources [13]. The Ministry of Public Health (MoPH), policy makers, advocacy groups, media, academics, healthcare implementing organizations, and donors rely on cancer data from IARC, which estimates cancer incidence in Afghanistan on the basis of data from cancer registries in neighboring countries [14]. In view of the pivotal role of the population‐based cancer registry, Afghanistan's National Cancer Control Program (NCCP) established the first population‐based cancer registry, Kabul Cancer Registry (KCR), in 2018 [15, 16].

This study provides a comprehensive analysis of population‐level cancer incidence estimates among Kabul residents newly diagnosed between 2018 and 2020, disaggregated by sex, cancer site/type, and age group. It also estimates the incidence of childhood cancers. Additionally, the study compares the incidence rates of the most common cancers with IARC estimates for Afghanistan and with data from regional cancer registries in neighboring countries. As the first large‐scale analysis of its kind in Afghanistan, this study addresses a critical gap in the country's cancer epidemiology, offering valuable insights to inform evidence‐based strategies for cancer prevention and control.

## 2 Materials and Methods

### 2.1 Setting

**FIGURE 1:**
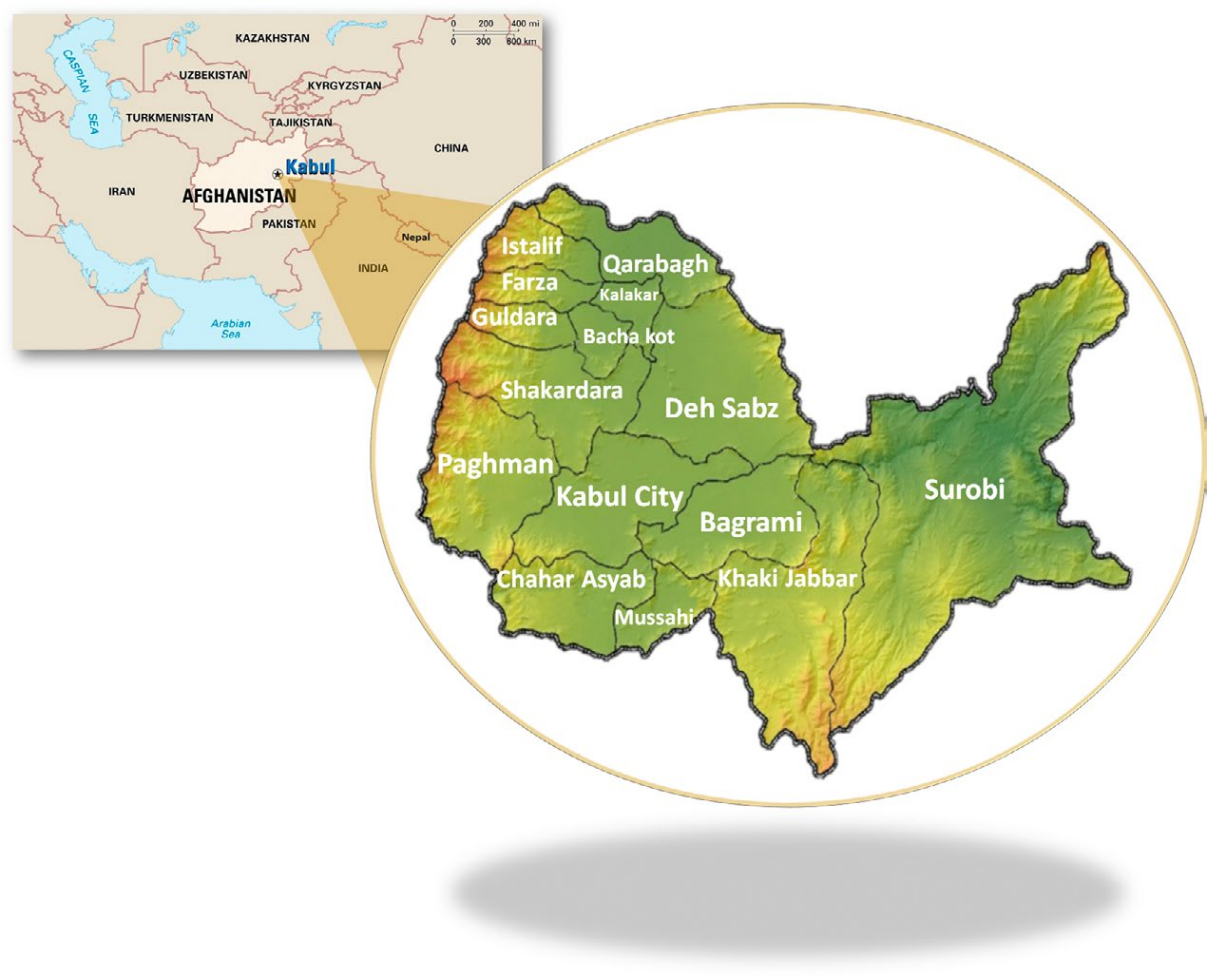
Map of Afghanistan showing its neighboring countries and a zoomed‐in view of Kabul Province with Kabul City and its 14 rural districts.

Among the 34 provinces in Afghanistan, Kabul is the most populated and urban province. Located in the eastern‐central part of the country, Kabul has been the cultural, political, and economic center in contemporary history [17]. With a land area of 4524 km (2811 sq. mil), it makes up only 0.7% of the total country area. The average population density of Kabul was calculated at 1112 persons per square kilometer in the years of study. For administrative purposes, Kabul Province was divided into 22 urban municipalities (Kabul city) and 14 rural districts (Figure 1). The vast majority of the Kabul population (85.0%) lived in Kabul city, while 15.0% lived in its rural districts [18]. People from all ethnic groups in the country, such as Pashtun, Tajik, Hazara, Uzbek, Turkmen, and others, lived in Kabul even though the proportion of each group differed [19]. There were 20 public tertiary hospitals and more than 50 private and not‐for‐profit tertiary hospitals in Kabul. In addition, there were two pathology laboratories in the public sector and approximately eight in the private sector in 2020.

### 2.2 The Kabul Cancer Registry

The KCR was established as a hospital‐based cancer registry in 2017 to register the cases of the cancer center in Jamhuriat Hospital. In 2018, it was expanded to a population‐based cancer registry within the NCCP. Located in Jamhuriat Hospital, KCR had an officer, three registrars, and an Information Technology officer [15]. The staff received multiple in‐person and online cancer registry trainings from the IARC hubs in Mumbai and Izmir as well as in Shaukat Khanum Memorial Cancer Hospital [16]. In collaboration with the Izmir Hub, a cancer registry abstract form, which was based on Afghanistan's context, was developed. The form contained 28 variables arranged in five sections. The mandatory variables included name, father name, age, sex, address, incidence date, topography, morphology, behavior, and basis of diagnosis [20].

### 2.3 Information Sources and Data Collection

Reporting of cancer cases was not legally mandated; however, the MoPH had requested health facilities to share cancer data with the cancer registry staff. The cancer registrars regularly visited 50 major sources where cancer was diagnosed and treated, including hospitals, pathology laboratories, imaging centers, and other health facilities in the public and private sectors in Kabul. The frequency of visits ranged from once a month to once every 3 months, depending on the patient volume and the number of cancer cases recorded at each facility. The staff manually abstracted information from paper‐based and electronic medical records on the abstract form. Once the forms were brought to the KCR office, they were checked again by other staff members. The information was subsequently entered into CanReg5, which is IARC's freely available software for entering, storing, checking, and processing cancer data [21]. Within CanReg5, cancer cases were entered and managed in two separate databases: one for Kabul residents and another for residents of other provinces.

Data for this study were extracted from KCR in March 2024. By that time, the KCR had documented a total of 4944 cancer cases with malignant behavior three (3) among Kabul residents diagnosed between 2018 and 2020. The majority of these cases (4071) were collected during the initial operational period of the registry, spanning January 2018 to August 2021. However, data collection was interrupted in August 2021 due to political changes in the country, which led to the dismantling of the KCR. Following the reestablishment of the registry and the appointment of a new KCR team, data collection resumed in October 2022. As of March 2024, an additional 873 cases had been abstracted and entered into the CanReg5 database. Of the total cases, nearly one‐third (31.9%) were reported by the French Medical Institute for Mothers and Children, and more than a quarter (27.0%) were reported by the cancer center at Jamhuriat Hospital, a public sector facility.

### 2.4 Definitions and Standards

The KCR followed the guidelines and standards established by the International Association of Cancer Registries (IACR) and the IARC. For example, patients who had resided in Kabul for at least 6 months prior to their cancer diagnosis were classified as residents of Kabul province. Patients who had lived in Kabul for < 6 months or had temporarily moved to Kabul for cancer diagnosis or treatment were considered residents of their respective provinces of origin. The date of incidence was determined as the date the malignancy was first reported in the histology/cytology report (microscopically verified), confirmed by clinical investigations such as X‐ray, CT scan, MRI, mammography, endoscopy, ultrasound, and laboratory tests (excluding molecular and genetic tests) or diagnosed by clinical evaluation only [20]. The registry did not use date of death, date on the death certificate or date on the autopsy. Only cases with behavior/3 are included in the analysis. In addition, the registry collected data on carcinomas of the skin as well as malignant and benign tumors of the brain. For coding variables such as topography, morphology, and behavior, we used the International Classification of Disease for Oncology, third edition (ICD‐O‐3.2) [22]. For this study, we categorized the cancer sites on the basis of the ICD‐10 [23]. For childhood cancers, we applied the International Classification of Childhood Cancer, 3rd edition (ICCC‐3) [24].

### 2.5 Duplicates, Multiple Primaries and Data Validation

We utilized IARCcrg Tools, which adhere to the IARC/IACR rules, to check for duplicates, multiple primaries, and internal consistency [25]. Subsequently, we manually checked for duplicates using the patient's name and the father's name (in the national language), using Excel software. The matched cases were further filtered by address (province), topography, morphology, and phone number, as patients did not have a unique personal identifier in Afghanistan. Of the 4944 cases, 446 (9.0%) were reported by multiple facilities (sources) and subsequently excluded, resulting in a total of 4498 valid cases. No cases with multiple primary cancers were identified in this dataset. To ensure internal consistency, we used the IARC CHECK program to evaluate the following variables in accordance with IARC recommendations: age/incidence, age/site/histology, site/histology, sex/site, sex/histology, behavior/site, behavior/histology, grade/histology, and basis of diagnosis/histology [26].

### 2.6 The Kabul Population

**FIGURE 2:**
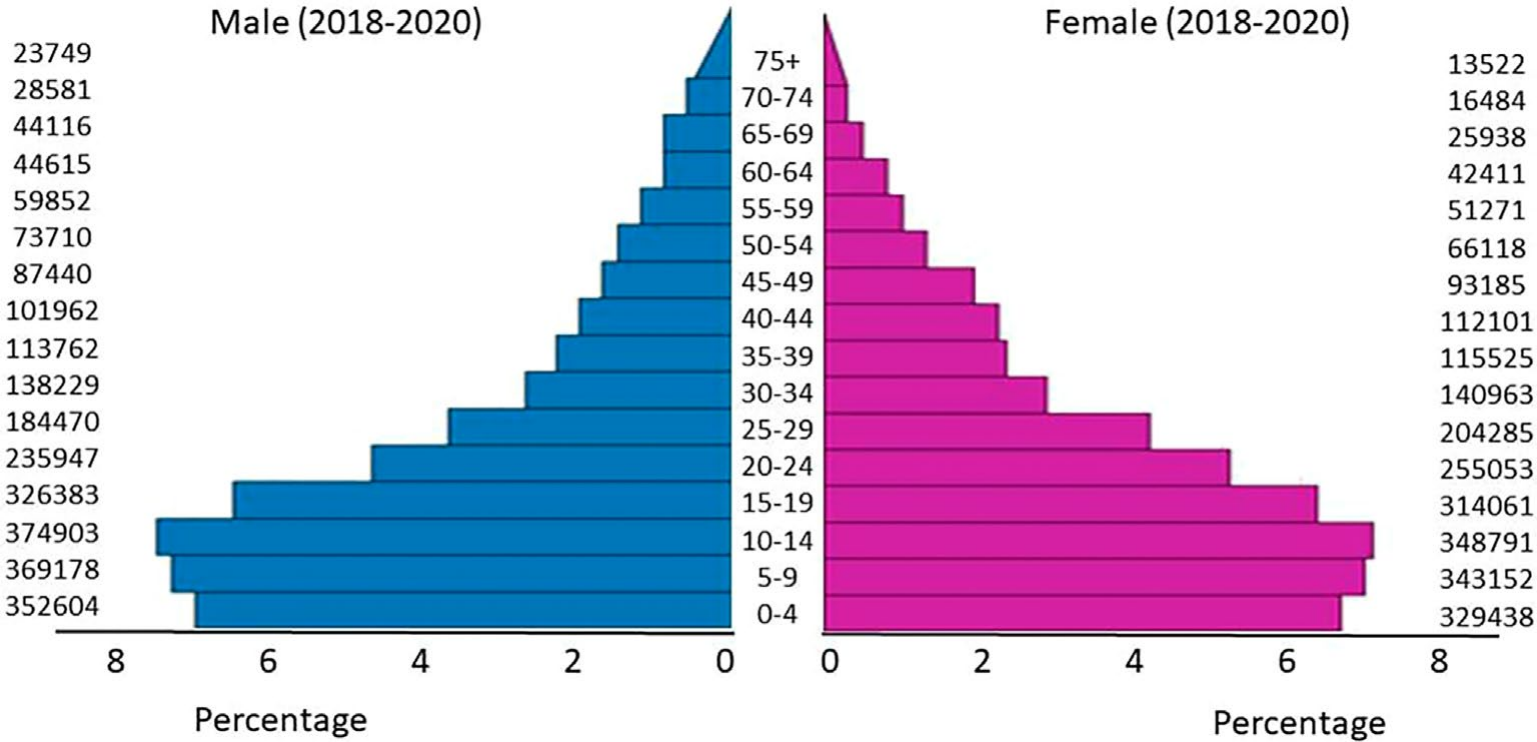
Population pyramid showing the average annual person‐years by sex and age group in Kabul, Afghanistan, 2018–2020.

The population of Kabul was estimated to be 4,860,880 in 2018, 5,029,850 in 2019, and 5,204,667 in 2020, accounting for approximately 15% of the country's total population. Since the last census was conducted in 1979, the National Statistics and Information Authority (NSIA) relied on the Household Listing Survey (HHL 2003–2005) to estimate the population for these years [18]. The annual average population during the study period (2018–2020) was calculated to be 5,031,799. To derive age‐specific population denominators, the population was extrapolated from the 14 age groups provided by the NSIA (0–4, 5–9, …, 65+) into three additional age groups (65–69, 70–74, 75+), based on corresponding population percentages supplied by the NSIA. The population pyramid (Figure 2) illustrates a skewed age distribution, with over 40% of the population under the age of 15 and only 3% aged 65 or older.

### 2.7 Incidence Rates Calculations

We computed crude, age‐specific, and age‐standardized incidence rates (ASIRs) for cancer sites by sex in Kabul. Using the available age‐specific population data, we divided the population into 16 age groups (0–4, 5–9, …, 75+). ASIRs were calculated using the direct method, with Segi's World Standard Population as the reference [27]. The incidence rates for the general population are expressed per 100,000 person‐years, while the incidence rates for childhood cancers are presented per 1,000,000 person‐years. We reported the most common cancers (by number of cases) and top cancers with the highest ASIRs, including nonmelanoma skin cancer (ICD‐10 code C44).

### 2.8 Data Analysis

We used CanReg5 version 5.00.44 g, Microsoft Excel 2016, and R version 4.4.0 for the analysis.

## 3 Results

### 3.1 Descriptive Statistics

Between 2018 and 2020, 4498 new cancer cases of Kabul residents were registered by the KCR. Among the total observed cases, 6.8% (*N* = 306) occurred in children (aged 0–14 years), 2.0% (*N* = 91) in adolescents (aged 15–19 years), 18.7% (*N* = 842) in adolescents and young adults (aged 15–39 years), and 25.9% (*N* = 1166) in older adults (aged ≥ 65 years). Females accounted for nearly 55% (*N* = 2468) of the cases, with a male‐to‐female ratio of 0.82. The age of the patients ranged between 0 and 99 years. The overall mean age (±SD) at the time of first diagnosis was reported to be 49.7 years (±19.5), with 52.5 years (±21.0) in males and 47.4 years (±17.8) in females. The estimated mean ages of the five most common cancers (by number of cases) are summarized in Table S1.

### 3.2 Most Common Cancers

Based on the available data, the most common cancers among males in Kabul were stomach cancer, accounting for 18.6% (*N* = 378) of all male cases, followed by esophageal, colorectal, other skin, lymphoma, and leukemia. Among females, breast cancer was the most commonly diagnosed, comprising 26.2% (*N* = 647) of all female cases, followed by esophageal, stomach, colorectal, leukemia, and other skin. When both sexes were combined, breast cancer remained the leading cancer, representing 14.9% (*N* = 668) of all cases, followed by stomach, esophageal, colorectal, other skin, and lymphoma, which together accounted for 52.4% (*N* = 2363) of all cancer cases (Table S1).

### 3.3 Incidence Rates

The age‐specific incidence rates in males were observed to increase with age, peaking at 345.3 per 100,000 person‐years in those aged 75 years or older, followed by 249.6 in those aged 70–74. Similarly, the rates in females were highest at 335.3 per 100,000 person‐years in patients aged 75 years or older, followed by 254.8 in those aged 70–74 (Tables S2 and S3).

The overall ASIR in males and females combined was estimated to be around 52.5 per 100,000 person‐years, which was 44.3 in males and 60.9 in females. In males, the five cancers with the highest ASIRs were reported to be stomach (ASIR = 9.1), esophagus (ASIR = 5.5), colorectum (ASIR = 3.5), lymphoma (ASIR = 2.4), and liver (ASIR = 2.1). In females, the five cancer sites with the highest ASIRs were estimated to be the breast (ASIR = 14.9), esophagus (ASIR = 6.7), stomach (ASIR = 4.2), colorectum (ASIR = 3.8), and gallbladder (ASIR = 1.8) (Table 1).

**TABLE 1:** Estimated crude and age‐standardized incidence rates (ASIRs) by cancer site and sex in Kabul, Afghanistan, 2018–2020.

Cancer site/type	ICD‐10	Males				Females			
		*N*	%	Crude	ASIR	*N*	%	Crude	ASIR
Lip	C00	0	0.0	0.0	0.0	0	0.0	0.0	0.0
Tongue	C01‐02	5	0.2	0.1	0.1	8	0.3	0.1	0.3
Mouth	C03‐06	14	0.7	0.2	0.3	5	0.2	0.1	0.2
Salivary gland	C07‐08	10	0.5	0.1	0.2	10	0.4	0.1	0.3
Tonsils	C09	1	0.0	0.0	0.0	0	0.0	0.0	0.0
Other oropharynx	C10	4	0.2	0.1	0.1	0	0.0	0.0	0.0
Nasopharynx	C11	4	0.2	0.1	0.1	0	0.0	0.0	0.0
Hypopharynx	C12‐13	1	0.0	0.0	0.0	1	0.0	0.0	0.0
Pharynx unspecified	C14	1	0.0	0.0	0.0	0	0.0	0.0	0.0
Esophagus	C15	221	10.9	2.9	5.5	217	8.8	2.9	6.7
Stomach	C16	378	18.6	4.9	9.1	148	6.0	2.0	4.2
Small intestine	C17	21	1.0	0.3	0.5	19	0.8	0.3	0.6
Colon	C18	88	4.3	1.1	2.0	78	3.2	1.1	2.2
Rectum	C19‐20	69	3.4	0.9	1.5	58	2.4	0.8	1.6
Anus	C21	7	0.3	0.1	0.2	10	0.4	0.1	0.2
Liver	C22	93	4.6	1.2	2.1	61	2.5	0.8	1.7
Gallbladder, etc.	C23‐24	45	2.2	0.6	1.1	64	2.6	0.9	1.8
Pancreas	C25	56	2.8	0.7	1.4	36	1.5	0.5	1.1
Nose, sinus, etc.	C30‐31	5	0.2	0.1	0.1	1	0.0	0.0	0.0
Larynx	C32	7	0.3	0.1	0.2	2	0.1	0.0	0.0
Trachea, bronchus, lung	C33‐34	23	1.1	0.3	0.5	22	0.9	0.3	0.6
Other thoracic organs	C37‐38	2	0.1	0.0	0.0	5	0.2	0.1	0.1
Bone	C40‐41	49	2.4	0.6	0.7	49	2.0	0.7	0.8
Melanoma of skin	C43	10	0.5	0.1	0.2	9	0.4	0.1	0.2
Other skin	C44	137	6.7	1.8	3.3	90	3.6	1.2	2.9
Mesothelioma	C45	0	0.0	0.0	0.0	1	0.0	0.0	0.0
Connective and soft tissue	C47, C49	31	1.5	0.4	0.6	31	1.3	0.4	0.5
Breast	C50	21	1.0	0.3	0.5	647	26.2	8.7	14.9
Vulva	C51	—	—	—	—	11	0.4	0.1	0.3
Vagina	C52	—	—	—	—	9	0.4	0.1	0.2
Cervix uteri	C53	—	—	—	—	45	1.8	0.6	1.2
Corpus uteri	C54	—	—	—	—	22	0.9	0.3	0.6
Uterus, unspecified	C55	—	—	—	—	44	1.8	0.6	1.1
Ovary	C56	—	—	—	—	78	3.2	1.0	1.7
Other female genital org.	C57	—	—	—	—	39	1.6	0.5	0.8
Placenta	C58	—	—	—	—	9	0.4	0.1	0.1
Penis	C60	1	0.0	0.0	0.0	—	—	—	—
Prostate	C61	47	2.3	0.6	1.2	—	—	—	—
Testis	C62	39	1.9	0.5	0.6	—	—	—	—
Other male genital org.	C63	1	0.0	0.0	0.0	—	—	—	—
Kidney	C64	87	4.3	1.1	1.8	60	2.4	0.8	1.3
Renal Pelvis	C65	0	0.0	0.0	0.0	1	0.0	0.0	0.0
Ureter	C66	0	0.0	0.0	0.0	0	0.0	0.0	0.0
Bladder	C67	44	2.2	0.6	1.1	16	0.6	0.2	0.5
Other urinary organs	C68	0	0.0	0.0	0.0	0	0.0	0.0	0.0
Eye	C69	3	0.1	0.0	0.0	6	0.2	0.1	0.1
Brain, nervous system	C70‐72	58	2.9	0.8	1.0	82	3.3	1.1	1.7
Thyroid	C73	10	0.5	0.1	0.2	34	1.4	0.5	0.7
Adrenal	C74	7	0.3	0.1	0.1	9	0.4	0.1	0.2
Other endocrine	C75	0	0.0	0.0	0.0	0	0.0	0.0	0.0
Hodgkin's disease	C81	26	1.3	0.3	0.4	22	0.9	0.3	0.4
NH lymphoma	C82‐85, C96	105	5.2	1.4	1.9	53	2.1	0.7	1.2
Multiple myeloma	C90	3	0.1	0.0	0.1	2	0.1	0.0	0.1
Lymphoid leukemia	C91	47	2.3	0.6	0.6	45	1.8	0.6	0.5
Myeloid leukemia	C92‐94	36	1.8	0.5	0.5	33	1.3	0.4	0.4
Leukemia, unspecified	C95	21	1.0	0.3	0.2	21	0.9	0.3	0.3
Other and unspecified	O&U	193	9.5	2.5	4.1	256	10.4	3.5	6.4
All sites	All	2031	100.0	26.5	44.3	2467	100.0	33.3	60.9
All sites but C44	AllbC44	1894	93.3	24.7	41.0	2377	96.4	32.0	58.0

*Note:* The color shades in Table 1 separate various cancers by systems and category.

In children (aged 0–14 years), the overall ASIR was reported as 48.7 per 1,000,000 person‐years, with the highest age‐specific rates (52.8) in infants and toddlers (aged 0–4 years) and the lowest age‐specific rates (42.8) in preadolescents (aged 10–14 years). The five cancers with the highest ASIRs were estimated to include leukemia (ASIR = 21.1 per 1,000,000 person‐years), lymphoma (ASIR = 6.4), renal tumors (ASIR = 5.6), Central Nervous System (CNS) neoplasms (ASIR = 3.0), and soft tissue sarcomas (ASIR = 2.6). Leukemia was observed to account for 43.5% (*N* = 133) of all cancer cases in children (Table 2).

**TABLE 2:** Estimated age‐standardized incidence rates (ASIR) per million of childhood cancer in Kabul, Afghanistan, 2018–2020.

	ICCC3	Number of cases						Rates per million				
		0–4	5–9	10–14	All	M/F	% total	0–4	5–9	10–14	crude	ASIR
	All	108	105	93	306	1.2	100.0	52.8	49.1	42.8	48.2	48.7
I	Leukemias	45	45	43	133	1.0	43.5	22.0	21.1	19.8	20.9	21.1
II	Lymphomas	7	21	14	42	2.2	13.7	3.4	9.8	6.4	6.6	6.4
III	CNS neoplasms	4	8	8	20	0.5	6.5	2.0	3.7	3.7	3.1	3.0
IV	Neuroblastoma	2	0	0	2	1.0	0.7	1.0	0.0	0.0	0.3	0.4
V	Retinoblastoma	6	0	0	6	1.0	2.0	2.9	0.0	0.0	0.9	1.1
VI	Renal tumors	23	8	0	31	1.1	10.1	11.2	3.7	0.0	4.9	5.6
VII	Hepatic tumors	3	0	1	4	1.0	1.3	1.5	0.0	0.5	0.6	0.7
VIII	Malignant bone tumors	1	7	9	17	1.8	5.6	0.5	3.3	4.1	2.7	2.4
IX	Soft tissue sarcomas	7	4	5	16	2.2	5.2	3.4	1.9	2.3	2.5	2.6
X	Germ cell tumors	5	3	1	9	3.5	2.9	2.4	1.4	0.5	1.4	1.5
XI–XII	Other	5	9	12	26	1.4	8.5	2.4	4.2	5.5	4.1	3.9

*Note:* Incidence of childhood cancer, classified according to the International Classification of Childhood Cancer (ICCC‐3) [24].

### 3.4 Quality of the Cancer Data

Among the 4498 cancer cases reported by the KCR, 80.6% were verified by microscopic (MV%) examinations (cytology and histology), whereas 19.4% were verified through clinical information. The proportion of microscopically verified cases was greater in females (82.9%) than in males (77.7%). The proportion of microscopically verified cases was highest for melanoma of the skin (100%), followed by those of the thyroid (97.7%) and breast (97.2%), whereas it was lowest for the pancreas (26.1%), brain and CNS (35.7%), and liver (40.3%). Table 3 shows the percentage of microscopically verified and clinically diagnosed cases of major cancers recorded by the KCR. Moreover, none of the cases were reported through a death certificate only (DCO) because the Vital Statistics Department was recently established and had not collected data on deaths. Furthermore, of the total cases, the age of 39 cases (0.87%) was unknown. Besides, the registry contained 83 (1.8%) cases with an unknown primary site (ICD‐O=C80.9) and 449 (10.0%) cases with other and unspecified (O&U) sites.

**TABLE 3:** Percentage of cases at the major sites that were registered on the basis of microscopic verification (MV) and clinical information in Kabul, Afghanistan, 2018–2020.

Cancer site	ICD‐10	No. cases	% MV	% Clinical
Mouth and pharynx	C00‐14	64	92.2	7.8
Esophagus	C15	438	87.2	12.8
Stomach	C16	526	82.3	17.7
Colon, rectum, anus	C18‐21	310	79.4	20.6
Liver	C22	154	40.3	59.7
Pancreas	C25	92	26.1	73.9
Larynx	C32	9	88.9	11.1
Trachea, bronchus, lung	C33‐34	45	77.8	22.2
Melanoma of skin	C43	19	100.0	0.0
Breast	C50	668	97.2	2.8
Cervix	C53	45	84.4	15.6
Corpus and uterus NOS	C54‐55	66	78.8	21.2
Ovary and adnexa	C56	76	75.0	25.0
Prostate	C61	47	83.0	17.0
Testis	C62	39	87.2	12.8
Kidney & urinary NOS	C64‐66,68	148	75.0	25.0
Bladder	C67	60	90.0	10.0
Brain and central nervous system	C70‐72	140	35.7	64.3
Thyroid	C73	44	97.7	2.3
Lymphoma	C81‐85,90,88,96	211	96.2	3.8
Leukemia	C91‐95	203	98.0	2.0
All sites	All	4498	80.6	19.4

## 4 Discussion

This paper presents findings from the initial three‐year (2018–2020) dataset of the KCR, which was established in 2018. Notably, this study provides the first estimates of cancer incidence in Kabul, the capital of Afghanistan. We compared the KCR data with IARC estimates for Afghanistan, which are based on the rates in neighboring countries—Pakistan, Tajikistan, and Uzbekistan [14]. Additionally, we compared the incidence rates (ASIRs) of the top ten cancers with the highest ASIRs in Kabul with those of the same cancers in Khyber Pakhtunkhwa (KPK) [28] and Lahore [29] in Pakistan, Delhi [30] in India, Kathmandu [31] in Nepal, and Razavi‐Khorasan and Sistan & Baluchestan [32] (S&B) in Iran (Table 4). Among these provinces/states/cities, KPK is geographically and culturally closest to Kabul, followed by Razavi‐Khorasan and S&B, which also share borders with Afghanistan.

**TABLE 4:** Estimated age‐standardized incidence rates (ASIRs) of the top 10 cancers in Kabul compared with other regions.

	ICD‐10	Kabul	Afghanistan (Globocan)	KPK^b^	Lahore^b^	Delhi^c^	Kathmandu^c^	Razavi Khorasan	S&B
Time Period		2018–2020	2022	2020	2010–2019	2012–2014	2018	2018	2018
Population		5,031,799	38,928,341	38,589,937	11,128,776	51,923,020	3,071,932	6,610,537	2,775,014
**ASIRS in males**									
Stomach	C16	9.1	14.0	4.4	2.6	3.8	8.5	24.1	8.5
Esophagus	C15	5.5	5.7	3.9	2.5	6.5	2.6	9.1	3.9
Colorectum	C18–20	3.5	6.1	5.8	7.0	7.5	6.8	21.5	0.8
Lymphoma	C81–85, C90, C96	2.4	4.3	8.6	9.5	14.7	6.2	8.5	5.4
Liver	C22	2.1	5.5	1.9	5.7	4.1	2.1	5.3	2.7
Kidney	C64	1.8	3.3	2.6	2.7	2.8	2.2	3.1	1.7
Leukemia	C91–C95	1.4	3.8	1.7	2.0	7.5	3.1	8.0	3.5
Pancreas	C25	1.4	2.3	1.3	1.0	2.4	2.7	4.1	1.1
Prostate	C61	1.2	6.5	6.6	10.7	11.8	3.0	11.6	4.7
Bladder	C67	1.1	4.1	0.8	8.0	6.8	5.7	7.2	5.4
**ASIRs in females**									
Breast	C50	14.9	29.4	27.9	76.7	38.6	21.5	34.6	17.5
Esophagus	C15	6.7	4.0	4.6	2.1	3.8	1.5	7.5	5.8
Stomach	C16	4.2	8.2	2.4	2.0	2.4	4.2	10.5	4.6
Colorectum	C18–C20	3.8	5.2	4.1	4.1	4.9	4.7^a^	16.0	5.8
Gallbladder	C23, C24	1.8	1.1	1.5	2.8	11.6	7.4	1.3	0.7
Ovary	C56	1.7	4.2	4.7	7.1	9.5	5.5	4.4	1.9
Corpus & uterus NOS	C54, C55	1.7	5.0	3.3	9.0	6.3	3.4	5.2	3.4
Liver	C22	1.7	3.7	1.2	3.7	2.0	0.8	4.0	1.8
Brain and CNS	C70–C72	1.7	3.2	1.2	3.7	2.8	1.7	7.2	2.1
Lymphoma	C81–85, C90, C96	1.7	3.0	4.8	7.1	10.0	4.1	5.1	3.1

Abbreviations: KPK, Khyber Pakhtunkhwa province in Pakistan; S&B, Sistan and Baluchistan province in Iran.

^a^ Colorectum and anus combined.

^b^ The ASIRs of KPK and Lahore are adults only.

^c^ The rates of Delhi and Kathmandu are age‐adjusted rates.

Based on our findings, the three most common cancers (by number of cases) among males in Kabul were observed to be stomach (18.6%), esophagus (10.9%), and colorectum (7.7%). Nationally, however, the leading cancers estimated by IARC in 2022 were stomach (11.3%), lung (9.1%), and oral cavity (6.1%) [14], highlighting some differences between local and national trends [33]. Among neighboring regions, only Razavi Khorasan and S&B closely aligned with Kabul, reporting stomach and colorectum as two of the most common cancers in males.

The three most common cancers (by number of cases) among females in Kabul were breast (26.2%), esophagus (8.8%), and stomach (6.0%). Breast cancer was the most common cancer among females in all cancer registries examined, including IARC's national estimates (27.2%) and neighboring regions; however, differences were noted in the ranking of other cancers. For example, IARC estimates that cervix uteri cancer accounted for 9.3% of all female cancers nationally, whereas our data suggest it constituted only 1.8% of the cases in Kabul. In neighboring registries, Razavi Khorasan and KPK showed some alignment, with stomach and esophagus cancer ranking as the third most common cancers, respectively (Table S4).

Overall, cancer was observed to be more common among females in Kabul, with an estimated male‐to‐female age‐standardized incidence rate ratio (ASIRR) of 0.7:1, consistent with national estimates for Afghanistan (ASIRR = 0.9:1) as reported by IARC [33]. This trend of cancer being more common in females was also observed in other cities and provinces, including Lahore (ASIRR = 0.6:1), Kathmandu (ASIRR = 0.96:1), S&B (ASIRR = 0.91:1), and KPK (ASIRR = 0.86:1). In contrast, Razavi‐Khorasan (ASIRR = 1.04:1) and Delhi (ASIRR = 1.04:1) were exceptions, where cancer incidence was slightly higher in males than females. Globally, cancer incidence was higher in males than females, with an ASIRR of 1.1:1.

The ASIRs for all cancers in Kabul (males = 44.3, females = 60.9) were reported to be lower than the national estimates provided by IARC (males = 110.7, females = 103.6). Similarly, the ASIRs for all cancers in Kabul were lower than those in Lahore (males = 103.1, females = 167.8), Razavi‐Khorasan (males = 154.7, females = 148.3), Delhi (males = 147.0, females = 141.0), Kathmandu (males = 86.7, females = 90.8), S&B (males = 75.1, females = 82.6), and KPK (males = 73.0, females = 84.9). The higher incidence of cancer in these regions could be attributed to exposure to various risk factors, particularly leading to higher incidences of cancer of the lip and oral cavity, stomach, lung, bladder, and prostate in males, and cancer of the breast, uterus, and ovary in females [34, 35].

The ASIR of female breast cancer in Kabul (14.9) appeared to be lower than the national estimate (29.4) and significantly lower than those reported in Lahore (76.7), Delhi (38.6), and Razavi Khorasan (34.6). It was moderately lower than the rates in KPK (27.9) and Kathmandu (21.5), and relatively lower than that in S&B (17.5). Globally, breast cancer was the leading cancer in 2020, accounting for nearly 12.0% of all cancer cases, with an ASIR of 47.8 per 100,000. Regionally, the highest rates were reported in Australia and New Zealand (ASIR = 95.5) and Western Europe (ASIR = 90.7), while the lowest rates were observed in South Central Asia (ASIR = 26.2) and Middle Africa (ASIR = 32.7) [36]. At the country level, the ASIR ranged from 105.4 per 100,000 in France to 4.6 per 100,000 in Bhutan [33].

The nonmodifiable risk factors (older age, family history, genetic mutations, pregnancy and breastfeeding, menstrual period and menopause, and density of breast tissue) and modifiable risk factors (overweight/obesity, physical inactivity, exposure to chemicals, estrogen, alcohol, and smoking) of breast cancer are well studied around the world [37, 38]. However, further research is needed to understand whether differences in the known risk factors explain the observed rates in Kabul. It is possible that the lower prevalence of certain risk factors, such as the use of oral contraceptives, alongside a higher prevalence of others, such as breastfeeding, may contribute to the differences observed compared to other cancer registries.

The ASIR of stomach cancer among males in Kabul (9.1) was reported to be lower than the national estimate (14.0) by IARC and that in Razavi Khorasan (24.1) but higher than in KPK (3.9), Lahore (1.6), Delhi (3.79), Kathmandu (8.3), and S&B (8.5). Globally, stomach cancer ranked as the fifth most common cancer in 2020, accounting for 5.6% of all cancer cases. The ASIR of stomach cancer in males was 15.8 worldwide, with the highest rates in East Asia (32.5) and the lowest rates in Middle Africa (4.6). The ASIRs of stomach cancer in males in Tajikistan (28.7) and Iran (22.4), both bordering Afghanistan, were among the highest in the world [39].

The development of stomach cancer is linked to various risk factors, including demographic, environmental, and lifestyle factors. These include advanced age, male sex, *H. pylori* infection, high consumption of salt and salted/smoked foods, low fruit and vegetable intake, low socioeconomic status, tobacco use, excessive alcohol consumption, sedentary lifestyle, obesity, and gastroesophageal reflux disease [40]. While there is a dearth of studies on risk factors for stomach cancer in Afghanistan, research focused on provinces with higher incidence rates in Iran, such as Razavi Khorasan, has identified *H. pylori* infection, high dietary salt intake, tobacco smoking, and obesity as the primary risk factors [41, 42]. Similarly, the main risk factors for stomach cancer in Pakistan are *H. pylori* , lower consumption of vegetables, and tobacco smoking [43]. While the prevalence of several risk factors such as alcohol consumption, obesity, and red meat consumption is lower in Afghanistan compared to neighboring countries, the intake of salt and salted foods is higher [44, 45].

The estimated ASIR of esophageal cancer among males in Kabul (5.5) was consistent with national estimates (5.7) by IARC; however, among females, the ASIR in Kabul (6.7) was notably higher than the national estimates (4.0). Compared to neighboring regions, the ASIRs in Kabul were higher than those reported in KPK (males = 3.9, females = 4.6), Lahore (males = 2.5, females = 2.1), Kathmandu (males = 2.6, females = 4.2), and Delhi (males = 6.5, females = 3.8), but lower than in Razavi Khorasan (males = 9.1, females = 7.5) and S&B (males = 3.8, females = 5.8).

Interestingly, the incidence of esophageal cancer in Kabul was 20% higher in females than in males (ASIRR = 0.8). This pattern of higher incidence in females was consistent with neighboring registries in S&B and Kathmandu, although it contrasts sharply with the global trend, where the ASIR of esophageal cancer was 2.5 times higher in males than females (ASIRR = 2.5). Globally, esophageal cancer was the eighth most common cancer in 2020, accounting for 3.0% of all cancer cases. The global ASIR for esophageal cancer was 6.3 per 100,000 persons, with significant regional variations. The highest rates were observed in Eastern Asia (12.0) and Eastern Africa (7.3), while the lowest rates were reported in Central America (0.9) and Western Africa (1.3). At the country level, the highest ASIRs among males were observed in Mongolia (20.7) and Malawi (20.3) [46].

The risk factors associated with esophageal cancer deaths in 2017 were tobacco smoking (39.0%), alcohol consumption (33.8%), overweight and obesity (19.5%), lower consumption of fruit and vegetables in the diet (19.1%), and the use of chewing tobacco (7.5%) [47]. A nonmatching case–control study (264 patients between Jan 2019 and Dec 2019) and a cross‐sectional study (240 patients between Jan 2019 and Feb 2021) in a pathology laboratory in Kabul identified several factors significantly associated with esophageal cancer. These included older age, rural residency, low or no educational attainment, snuff use, hot tea consumption, low physical activity, low fresh fruit consumption, being a farmer, and low economic status. In contrast, the study revealed no associations between esophageal cancer incidence and alcohol consumption, tobacco smoking, spicy food, pickled vegetables, or overweight/obesity. Unlike the findings from the KCR, males had a higher risk of esophageal cancer than females in the two studies. However, the participants in those studies were not limited to residents of Kabul, suggesting differences in population demographics [48, 49].

Surprisingly, our study revealed that lip and oral cavity, lung, and prostate cancers were relatively uncommon in Kabul but common in the region. The ASIRs of lip and oral cavity cancers in Kabul (males = 0.3, females = 0.4) were significantly lower than the IARC estimates for Afghanistan (male = 6.3, female = 3.0) and those reported in KPK (males = 3.9, females = 2.4), Lahore (males = 6.8, females = 5.7), Delhi (males = 19.7, females = 6.4), Kathmandu (males = 5.9, females = 1.8), Razavi Khorasan (males = 1.8, females = 2.1), and S&B (males = 2.4, females = 2.5). The higher consumption of alcohol and tobacco, especially smokeless tobacco with/without betel quid/pan, gutka, and areca nut, in South Asia could be attributed to the higher incidence rates of oral cavity cancers in the region [50, 51].

Similarly, our estimates indicate that the ASIRs of lung cancer in Kabul (males = 0.5, females = 0.3) were substantially lower than the IARC estimates for Afghanistan (males = 11.0, females = 3.6) and those reported in KPK (males = 2.5, females = 1.9), Lahore (males = 7.6, females = 2.3), Delhi (males = 16.7, females = 5.1), Kathmandu (males = 18.1, females = 10.4), Razavi Khorasan (males = 12.0, females = 7.2), and S&B (males = 3.2, females = 1.8). The higher prevalence of tobacco smoking in KPK (8.9%), Lahore (10.5%) [52], Delhi (11.3%) [53], Kathmandu (17.1% Nepal) [54], Razavi Khorasan (17.0%), and S&B (18.5%) [55] than in Kabul (8.1%) [56] could be associated with higher ASIRs of lung cancer. While tobacco smoking has been a well‐established cause of lung cancer incidence and death for decades [57, 58], new studies have shown an increase in lung cancer in never smokers (LCINS), particularly in developing countries. Proposed risk factors for LCINS include exposure to radon, asbestos, cooking fumes, heavy metals, environmental tobacco smoke, inherited genetic susceptibility, and human papillomavirus infection [59, 60, 61].

In children, the estimated incidence rates in Kabul were lower compared to the lowest deciles of childhood cancer incidence rates reported for high quality data [26]. However, the overall ASIR (48.7/million) was comparable to the rates in KPK (51.0/million) but lower than in Lahore (boys = 89.0/million; girls = 61.0/million). We observed that renal tumors ranked third highest in Kabul after leukemias and lymphomas; however, they ranked much lower in KPK and Lahore [28, 29].

Recent projections have indicated that global cancer cases will rise disproportionately by 2050, with the highest increases expected in low‐income countries (142.1%). Afghanistan is projected to experience a 154.6% (males and females combined) increase in cancer cases—a rate exceeding those of neighboring countries, including Iran (135.3%), Pakistan (107.3%), India (90.4%), Nepal (92.0%), Tajikistan (131.6%), and Uzbekistan (88.4%). However, these projections were based on GLOBOCAN data [62]. To improve the accuracy of future projections and to develop effective prevention strategies, it is essential to generate comprehensive cancer registry data and to better understand local trends in cancer incidence and risk factors—both of which remain limited in Afghanistan.

Like other cancer registry data from low‐income countries, our study has certain limitations [20]. One key limitation was the inclusion of the inaugural dataset from 2018, which reported 28.2% fewer cases than those reported in subsequent years (2019 and 2020). Despite active data collection by registry staff from approximately 50 major sources, the nonmandatory nature of cancer reporting likely led to underreporting, with some cases potentially not being captured. Additionally, the absence of a vital statistics system complicated the inclusion of cancer cases identified solely through DCO reports, resulting in an underestimation of cancer incidence, particularly for cancers with poor survival rates. Also, we used the estimated population data from NSIA due to the absence of a recent national census. However, these estimates have a demographic structure similar to the 1979 census data.

Furthermore, while many cancer registries globally, particularly in low‐income countries, experienced significant disruptions due to the Covid‐19 pandemic [63], the KCR saw only minimal disruption, with a 1.5% reduction in case registrations in 2020 compared to 2019. This resilience can be attributed to several factors, including stable funding and staffing, as well as the continued operation of key healthcare facilities, such as the cancer center at Jamhuriat Hospital [64], which allowed the registry to maintain active data collection. Nevertheless, despite these efforts, the broader healthcare disruptions during the pandemic may have still influenced the completeness and timeliness of cancer diagnoses and treatment reporting, potentially affecting the quality of the registry data. Further research is needed to assess the long‐term impact of the pandemic on cancer registration and care in Afghanistan.

We evaluated the quality of data from the KCR across four key dimensions: comparability, validity, timeliness, and completeness, noting alignment with registries in low‐income regions, particularly in Central, East, and South Asia (CESA). The KCR follows international standards set by IARC/IACR for comparability, including defining malignancies, recording the date of incidence, and using ICD‐10 and ICD‐O codes for topography, morphology, behavior, grade, and multiple primaries.

For validity, the proportion of microscopically verified cases (MV%) was within the acceptable range for all cancers combined (males: 77.7%, females: 82.6%). However, MV% varied by cancer type, being lower for cancers like pancreas and brain & CNS, and higher for liver and O&U cancers. The proportion of O&U cases (males: 10.2%, females: 10.8%) was acceptable for low‐income countries but slightly exceeded the < 10% threshold for high‐quality registries. Notably, no cases were identified through DCO as KCR did not collect death certificate data.

Timeliness was assessed by the interval from diagnosis to data entry into CanReg5, ranging from 6 to 18 months—comparable to other resource‐limited registries. However, operations were interrupted during the governmental transition (2021–2022), causing additional delays and reflecting challenges faced in conflict‐affected settings.

Completeness remains a challenge for the relatively young KCR. Nevertheless, the ASIRs for 2019 and 2020 showed stability, with no marked fluctuations in most cancers. For instance, ASIRs for all cancers excluding nonmelanoma skin cancers (C44) in both sexes combined remained consistent (2019 = 53.5, 2020 = 51.7), as did rates for breast cancer in females (2019: 15.5; 2020: 17.0) and digestive organ cancers (2019 = 24.0, 2020 = 24.5). However, the absence of mortality‐to‐incidence ratio (M:I%) data, a key indicator of completeness, remains a limitation that highlights the need for enhanced data collection and mortality linkage efforts [20, 26].

Moreover, factors such as low socioeconomic status, stigma surrounding cancer, low levels of education and awareness about cancer, an inadequate referral system, and insufficient specialized health centers may have contributed to cases being undiagnosed and unrecorded [65]. For instance, the country had merely one specialized chest hospital and one stomatology hospital in the public sector (MoPH), which could be attributed to the very low cancer cases of lung and lip/oral cavity cancers, respectively. Furthermore, some cancer patients sought diagnosis and treatment abroad, leading to incomplete data capture even though 6.4% of the cases in the KCR database were identified as such.

Regional comparisons, a semi‐quantitative method for assessing the completeness of cancer registry data, revealed that cancer incidence rates (ASIRs) in Kabul were lower than those in neighboring regions. However, site‐specific rates for some cancers were comparable to those in S&B in Iran and KPK in Pakistan. While some ethnic, cultural, and geographic similarities may explain these overlaps, Afghanistan's unique socioeconomic, environmental, and lifestyle contexts—shaped by nearly five decades of ongoing conflict and crises—likely contributed to the divergent cancer incidence rates observed in Kabul. Moreover, IARC's cancer incidence estimates for Afghanistan, derived from the mean rates of Uzbekistan, Tajikistan, and Pakistan, are higher than those reported by the KCR. This discrepancy underscores the need for locally derived data to more accurately reflect Afghanistan's cancer burden.

The KCR provides valuable insights into the cancer patterns of a historically underserved population, filling a significant gap in the global cancer registry network. It plays a critical role in addressing disparities in cancer care and outcomes between Afghanistan and its regional neighbors, serving as a vital reference for improving cancer surveillance in countries facing similar challenges. As the registry matures, it will enhance the ability to track cancer trends, evaluate the effectiveness of public health interventions, and shape evidence‐based healthcare policies. Beyond Afghanistan, the KCR's findings contribute to the global cancer control agenda by highlighting the cancer burden in conflict‐affected, resource‐limited regions. These data not only address disparities in access to cancer care but also encourage international collaborations aimed at strengthening cancer surveillance infrastructure in low‐income and crisis‐affected settings, which is crucial for achieving global equity in cancer prevention, diagnosis, and treatment [66].

## 5 Conclusions

This pioneering study represents a vital milestone in cancer research for Afghanistan, particularly in Kabul, by offering the first population‐based insights into cancer incidence patterns. Despite inherent challenges in data collection and completeness, these findings provide a robust baseline for tracking cancer trends and conducting future comparisons. They underscore the urgent need for strengthening cancer registration and enhancing the data quality of KCR data.

By illuminating the epidemiology of major cancers, this research equips key stakeholders, including the MoPH, with critical evidence to formulate targeted strategies for primary prevention, early diagnosis, and improved treatment services. Additionally, this study highlights the potential of population‐based cancer registries in shaping health policy and guiding resource allocation in resource‐constrained settings.

As the first comprehensive report of its kind, this work not only contributes to addressing cancer disparities in underserved populations but also serves as a benchmark for the region and countries facing similar socio‐political and health system challenges. The findings align with global efforts to combat cancer and reinforce the essential role of registries in driving data‐informed, equitable healthcare interventions worldwide.

## Author Contributions

**Maihan Abdullah:** conceptualization (equal), formal analysis (equal), writing – original draft (equal). **Nisar Niazi:** formal analysis (equal), software (equal), visualization (equal), writing – review and editing (equal). **Goodarz Danaei:** conceptualization (equal), writing – review and editing (equal). **Jesse Bump:** conceptualization (equal), writing – review and editing (equal). **Timothy R. Rebbeck:** writing – review and editing (equal). **Ikram Hashimi:** writing – review and editing (equal). **Sabira Niyazi:** formal analysis (equal), visualization (equal). **Marwa Sarwari:** formal analysis (equal), visualization (equal). **Fatemeh Ghasemi‐Kebria:** methodology (equal), writing – review and editing (equal). **Gholamreza Roshandel:** methodology (equal), writing – review and editing (equal).

## Ethics Statement

Analysis of the KCR data was exempted by the Institutional Review Board (IRB) of the Afghanistan National Public Health Institute (ANPHI) within the Ministry of Public Health, with reference number A.0222.413.

## Conflicts of Interest

The authors declare no conflicts of interest.

## Supporting information


Data S1.

## Data Availability

Further information is available from the corresponding author upon reasonable request.
