# Supplementary material for: Cancer Incidence in Kabul, Afghanistan: The First Report From the Population‐Based Cancer Registry
Source: Cancer Med. 2025 Apr 18;14(8):e70844. doi: 10.1002/cam4.70844 (PMC12007459; doi:10.1002/cam4.70844)
Supplement: Supplementary file 1 — Data S1. [file CAM4-14-e70844-s001.docx]

**Cancer Incidence in Kabul, Afghanistan: The First Report from the Population-Based Cancer Registry**

**Cite as:**

**Supplementary Tables and Figures**

1. **Table S1.** Six most common cancer sites (by number of cases) among males, females, and both sexes combined, estimated by number, percentage, ASIR, and mean age with 95% confidence interval in Kabul, Afghanistan, 2018–2020.
2. **Table S2.** Age-specific incidence rates (per 100,000 population) of cancer sites, estimated among males in Kabul, Afghanistan, 2018-2020.
3. **Table S3.** Age-specific incidence rates (per 100,000 population) of cancer sites, estimated among females in Kabul, Afghanistan, 2018-2020.
4. **Table S4.** Comparison of the three most common cancers (by number of cases) among males and females in Kabul with Afghanistan, registries in neighboring countries, Asian regions, Asia, and global estimates.

| **Table S1**. Six most common cancer sites (by number of cases) among males, females, and both sexes combined, estimated by number, percentage, ASIR, and mean age with 95% confidence interval in Kabul, Afghanistan, 2018–2020. | | | | | | | | |
| --- | --- | --- | --- | --- | --- | --- | --- | --- |
| **Sex** | **Rank** | **Site** | **ICD-10** | **N** | **(%)** | **ASIR** | **Mean Age** | **95% CI** |
| **Males** | 1. | Stomach | C16 | 378 | 18.6 | 9.1 | 61.7 | 60.4 - 63.0 |
|  | 2. | Esophagus | C15 | 221 | 10.9 | 5.5 | 63.2 | 61.6 - 64.8 |
|  | 3. | Colorectum | C18-20 | 157 | 7.7 | 3.5 | 62.5 | 51.5 - 56.2 |
|  | 4. | Other skin | C44 | 137 | 6.7 | 3.3 | 52.5 | 49.8 - 55.2 |
|  | 5. | Lymphoma | C81-85, C90, C96 | 134 | 6.6 | 2.4 | 36.9 | 33.0 - 40.7 |
|  | 6. | Leukemia | C91-95 | 104 | 5.1 | 1.4 | 20.2 | 15.9 - 24.5 |
| **Females** | 1. | Breast | C50 | 647 | 26.2 | 14.9 | 46.8 | 45.8 - 47.7 |
|  | 2. | Esophagus | C15 | 217 | 8.8 | 6.7 | 57.3 | 55.6 - 59.0 |
|  | 3. | Stomach | C16 | 148 | 6.0 | 4.2 | 55.0 | 52.4 - 56.7 |
|  | 4. | Colorectum | C18-20 | 136 | 5.6 | 3.8 | 53.6 | 51.0 - 56.1 |
|  | 5. | Leukemia | C91-95 | 99 | 4.0 | 1.2 | 15.4 | 12.3 -18.4 |
|  | 6. | Other skin | C44 | 90 | 3.6 | 2.9 | 58.8 | 55.5 - 62.1 |
| **Males and Females** | 1. | Breast | C50 | 668 | 14.9 | 7.6 | 47.1 | 46.1 - 48.0 |
|  | 2. | Stomach | C16 | 526 | 11.6 | 6.7 | 60.0 | 58.5 - 60.9 |
|  | 3. | Esophagus | C15 | 438 | 9.7 | 6.1 | 60.0 | 59.1 - 62.0 |
|  | 4. | Colorectum | C18-20 | 293 | 6.5 | 3.7 | 54.0 | 52.0 - 55.5 |
|  | 5. | Other skin | C44 | 227 | 5.0 | 3.1 | 61.0 | 58.9 - 63.1 |
|  | 6. | Lymphoma | C81-85, C90, C96 | 211 | 4.7 | 2.0 | 37.3 | 34.2 - 40.4 |
| N: number of cases; (%): percentage of all cancers cases including other skin (C44); ASIR: age standardized incidence rate; 95% CI: 95% confidence interval. | | | | | | | | |

| **Table S2.** Age-specific incidence rates (per 100,000 population) of cancer sites, estimated among males in Kabul, Afghanistan, 2018-2020. | | | | | | | | | | | | | | | | | | |
| --- | --- | --- | --- | --- | --- | --- | --- | --- | --- | --- | --- | --- | --- | --- | --- | --- | --- | --- |
| **Site** | **ICD-10** | **(%)** | **0-** | **5-** | **10-** | **15-** | **20-** | **25-** | **30-** | **35-** | **40-** | **45-** | **50-** | **55-** | **60-** | **65-** | **70-** | **75-** |
| Lip | C00 | 0.0 | - | - | - | - | - | - | - | - | - | - | - | - | - | - | - | - |
| Tongue | C01-02 | 0.3 | - | - | - | - | - | - | 0.2 | - | - | - | 0.5 | 0.6 | 0.7 | 0.8 | - | - |
| Mouth | C03-06 | 0.7 | - | - | 0.1 | 0.2 | - | - | - | 0.3 | - | 0.4 | 0.9 | - | 3.0 | - | 1.2 | 2.8 |
| Salivary gland | C07-08 | 0.5 | - | - | - | 0.2 | - | - | 0.2 | - | 0.3 | 0.4 | - | - | - | 0.8 | 3.5 | 1.4 |
| Tonsils | C09 | 0.1 | - | - | - | - | - | - | - | 0.3 | - | - | - | - | - | - | - | - |
| Other oropharynx | C10 | 0.2 | - | - | - | - | - | - | - | - | 0.3 | 0.4 | - | - | - | 0.8 | 1.2 | - |
| Nasopharynx | C11 | 0.2 | - | - | 0.1 | - | - | - | - | 0.3 | - | 0.4 | - | 0.6 | - | - | - | - |
| Hypopharynx | C12-13 | 0.1 | - | - | - | - | - | - | - | - | - | - | - | - | - | - | 1.2 | - |
| Pharynx unspecified | C14 | 0.1 | - | - | - | - | - | - | - | - | - | - | - | - | - | - | - | 1.4 |
| Esophagus | C15 | 11.7 | - | - | - | - | 0.1 | 0.2 | 1.0 | 0.9 | 2.9 | 1.5 | 8.6 | 11.7 | 32..9 | 26.4 | 46.7 | 56.1 |
| Stomach | C16 | 20.0 | 0.1 | 0.1 | - | - | 0.3 | 0.7 | 1.0 | 2.1 | 4.2 | 6.5 | 17.6 | 28.4 | 41.8 | 47.6 | 70.0 | 84.2 |
| Small intestine | C17 | 1.1 | - | - | - | - | - | 0.2 | 0.7 | - | 0.3 | - | 2.7 | 1.1 | 3.0 | 0.8 | 1.2 | 2.8 |
| Colon | C18 | 4.6 | - | - | - | - | 0.4 | 0.7 | 0.5 | 1.8 | 1.0 | 2.7 | 5.9 | 6.1 | 12.0 | 5.3 | 7.0 | 14.0 |
| Rectum | C19-20 | 3.6 | - | - | - | - | 0.4 | 0.5 | 0.2 | 1.8 | 3.6 | 1.9 | 1.8 | 3.3 | 7.5 | 7.6 | 8.2 | 4.2 |
| Anus | C21 | 0.4 | - | - | - | - | - | - | - | - | 0.3 | - | 0.9 | - | 0.7 | - | 3.5 | - |
| Liver | C22 | 4.9 | 0.1 | - | 0.1 | - | 0.3 | 0.5 | 1.0 | 0.3 | 1.6 | 2.7 | 3.6 | 10.0 | 7.5 | 11.3 | 8.2 | 15.4 |
| Gallbladder etc. | C23-24 | 2.4 | - | - | - | - | - | - | 0.7 | 0.6 | 0.7 | 0.4 | 1.8 | 3.3 | 4.5 | 6.8 | 2.3 | 14.0 |
| Pancreas | C25 | 3.0 | - | - | - | - | - | - | 0.5 | 0.3 | 1.3 | 0.8 | 3.2 | 2.8 | 9.7 | 7.6 | 7.0 | 8.4 |
| Nose, sinus, etc. | C30-31 | 0.3 | - | - | - | - | - | - | 0.5 | - | - | 0.4 | - | - | 0.7 | 0.8 | - | - |
| Larynx | C32 | 0.4 | - | - | - | - | - | - | - | - | - | - | 0.9 | 1.1 | 0.7 | - | 2.3 | - |
| Trachea, bronchus, lung | C33-34 | 1.2 | - | - | - | - | - | 0.2 | 0.2 | - | 0.7 | - | 1.4 | 1.1 | 3.0 | 3.8 | 2.3 | 4.2 |
| Other thoracic organs | C37-38 | 0.1 | - | - | - | - | - | - | 0.2 | - | - | - | 0.5 | - | - | - | - | - |
| Bone | C40-41 | 2.6 | 0.2 | 0.5 | 0.6 | 1.2 | 0.1 | 0.5 | 0.5 | 0.6 | 1.0 | 0.4 | 1.4 | - | 3.0 | 0.8 | 2.3 | 1.4 |
| Melanoma of Skin | C43 | 0.5 | - | - | - | - | 0.1 | - | 0.5 | - | 0.3 | - | - | 0.6 | - | 2.3 | - | 2.8 |
| Other skin | C44 | 7.2 | - | - | - | 0.1 | 0.3 | 0.5 | 0.5 | 1.2 | 2.0 | 2.7 | 5.4 | 7.2 | 11.2 | 18.1 | 15.2 | 49.1 |
| Mesothelioma | C45 | 0.0 | - | - | - | - | - | - | - | - | - | - | - | - | - | - | - | - |
|  |  |  |  |  |  |  |  |  |  |  |  |  |  |  |  |  |  |  |
| Connective and soft tissue | C47, C49 | 1.6 | 0.3 | 0.2 | 0.2 | 0.1 | 0.3 | 0.4 | 0.5 | - | 0.3 | 0.4 | 1.4 | 1.7 | 3.7 | 0.8 | - | 4.2 |
| Breast | C50 | 1.1 | - | - | - | - | - | - | - | 0.9 | 0.3 | 1.9 | 1.4 | 0.6 | 1.5 | 0.8 | 3.5 | 2.8 |
| Penis | C60 | 0.1 | - | - | - | - | - | - | - | - | - | - | - | - | - | - | - | 1.4 |
| Prostate | C61 | 2.5 | - | - | - | - | - | - | - | 0.3 | - | 0.4 | 0.5 | 1.7 | 2.2 | 10.6 | 14.0 | 16.8 |
| Testis | C62 | 2.1 | 0.5 | 0.1 | - | - | 0.4 | 1.8 | 1.0 | 0.9 | 1.0 | 0.8 | 0.5 | 2.8 | - | 0.8 | - | 1.4 |
| Other male genital org. | C63 | 0.1 | - | - | - | - | - | - | - | - | - | - | - | - | - | 0.8 | - | - |
| Kidney | C64 | 4.6 | 1.3 | 0.3 | - | - | 0.1 | 0.4 | 0.7 | 0.3 | 1.0 | 3.4 | 5.4 | 7.2 | 3.0 | 3.8 | 11.7 | 9.8 |
| Renal Pelvis | C65 | 0.0 | - | - | - | - | - | - | - | - | - | - | - | - | - | - | - | - |
| Ureter | C66 | 0.0 | - | - | - | - | - | - | - | - | - | - | - | - | - | - | - | - |
| bladder | C67 | 2.3 | - | 0.2 | - | - | - | - | 0.2 | - | 0.3 | 1.1 | 1.4 | 4.5 | 8.2 | 5.3 | 2.3 | 8.4 |
| Other urinary organs | C68 | 0.0 | - | - | - | - | - | - | - | - | - | - | - | - | - | - | - | - |
| Eye | C69 | 0.2 | 0.3 | - | - | - | - | - | - | - | - | - | - | - | - | - | - | - |
| Brain, nervous system | C70-72 | 3.1 | 0.1 | 0.4 | 0.3 | 0.4 | 0.8 | 0.7 | 1.2 | 0.9 | 1.0 | 2.7 | 1.4 | 2.8 | 6.0 | 0.8 | 1.2 | - |
| Thyroid | C73 | 0.5 | - | - | - | - | - | 0.7 | 0.2 | 0.3 | 0.3 | 0.8 | - | - | - | 0.8 | - | - |
| Adrenal | C74 | 0.4 | - | - | 0.1 | - | - | - | 0.2 | 0.3 | - | - | - | - | 0.7 | 0.8 | - | 2.8 |
| Other endocrine | C75 | 0.0 | - | - | - | - | - | - | - | - | - | - | - | - | - | - | - | - |
| Hodgkin lymphoma | C81 | 1.4 | 0.1 | 0.5 | 0.3 | 0.3 | 0.1 | 0.5 | 0.5 | - | 0.7 | 0.8 | - | 0.6 | - | - | 1.2 | 2.8 |
| Non-Hodgkin lymphoma | C82-85,C96 | 5.5 | 0.5 | 0.9 | 0.4 | 0.5 | 1.3 | 1.3 | 1.4 | 1.2 | 2.6 | 3.8 | 1.4 | 2.8 | 2.5 | 6.0 | 3.5 | 4.2 |
| Multiple myeloma | C90 | 0.2 | - | - | - | - | - | - | - | - | - | - | 0.9 | 0.6 | - | - | - | - |
| Lymphoid leukemia | C91 | 2.5 | 1.0 | 1.2 | 0.6 | 0.5 | - | 0.2 | - | 0.3 | - | 0.4 | - | - | 2.2 | - | 3.5 | 2.8 |
| Myeloid leukemia | C92-94 | 1.9 | 0.5 | 0.5 | 0.8 | 0.1 | 0.1 | - | 0.5 | 0.6 | 0.3 | 0.8 | 0.9 | - | 1.5 | 1.5 | 1.2 | - |
| Leukemia, unspecified | C95 | 1.1 | 0.7 | 0.5 | 0.4 | - | 0.1 | 0.2 | 0.2 | 0.3 | 0.3 | - | - | - | - | - | - | - |
| Other and unspecified | O&U | 10.2 | 0.1 | 0.3 | 0.4 | 0.5 | 1.6 | 1.3 | 2.7 | 2.9 | 3.3 | 5.7 | 7.7 | 9.5 | 18.7 | 12.8 | 24.5 | 25.3 |
| All sites | All |  | 5.7 | 5.4 | 4.4 | 4.2 | 7.1 | 11.6 | 17.8 | 19.3 | 32.0 | 44.2 | 79.6 | 112.5 | 200.2 | 186.6 | 249.6 | 345.3 |
| All sites but C44 | AllbC44 | 100.0 | 5.7 | 5.4 | 4.4 | 4.1 | 6.8 | 11.0 | 17.4 | 18.2 | 30.1 | 41.6 | 74.2 | 105.3 | 189.0 | 168.5 | 234.4 | 296.1 |

| **Table S3.** Age-specific incidence rates (per 100,000 population) of cancer sites, estimated among females in Kabul, Afghanistan, 2018-2020. | | | | | | | | | | | | | | | | | | |
| --- | --- | --- | --- | --- | --- | --- | --- | --- | --- | --- | --- | --- | --- | --- | --- | --- | --- | --- |
| **Site** | **ICD-10** | **(%)** | **0-** | **5-** | **10-** | **15-** | **20-** | **25-** | **30-** | **35-** | **40-** | **45-** | **50-** | **55-** | **60-** | **65-** | **70-** | **75-** |
| Lip | C00 | 0.0 | - | - | - | - | - | - | - | - | - | - | - | - | - | - | - | - |
| Tongue | C01-02 | 0.3 | - | - | - | - | - | - | - | - | 0.6 | - | 0.5 | - | 0.8 | 1.3 | 2.0 | 4.9 |
| Mouth | C03-06 | 0.2 | - | - | - | - | - | - | - | - | - | 0.4 | - | 0.7 | 0.8 | 1.3 | - | 2.5 |
| Salivary gland | C07-08 | 0.4 | - | - | - | - | - | - | - | 0.6 | - | 0.4 | 2.0 | - | - | - | 2.0 | 4.9 |
| Tonsils | C09 | 0.0 | - | - | - | - | - | - | - | - | - | - | - | - | - | - | - | - |
| Other oropharynx | C10 | 0.0 | - | - | - | - | - | - | - | - | - | - | - | - | - | - | - | - |
| Nasopharynx | C11 | 0.0 | - | - | - | - | - | - | - | - | - | - | - | - | - | - | - | - |
| Hypopharynx | C12-13 | 0.0 | - | - | - | - | - | - | - | - | - | - | - | - | 0.8 | - | - | - |
| Pharynx unspecified | C14 | 0.0 | - | - | - | - | - | - | - | - | - | - | - | - | - | - | - | - |
| Esophagus | C15 | 9.1 | - | - | - | - | 0.1 | 0.2 | 0.9 | 1.7 | 6.8 | 7.2 | 15.1 | 15.6 | 21.2 | 46.3 | 40.4 | 61.6 |
| Stomach | C16 | 6.2 | - | - | - | - | 0.1 | 1.1 | 1.2 | 2.0 | 2.1 | 5.4 | 10.6 | 17.6 | 14.9 | 16.7 | 26.3 | 32.0 |
| Small intestine | C17 | 0.8 | - | - | - | - | - | 0.2 | - | - | 0.9 | 1.1 | - | 2.6 | 1.6 | 1.3 | 4.0 | 7.4 |
| Colon | C18 | 3.3 | - | - | - | 0.1 | 0.3 | 1.0 | 0.2 | 1.2 | 1.8 | 2.1 | 4.5 | 6.5 | 7.1 | 15.4 | 14.2 | 12.3 |
| Rectum | C19-20 | 2.4 | - | - | - | - | 0.4 | 0.5 | 0.7 | - | 0.9 | 2.5 | 2.5 | 4.6 | 9.4 | 9.0 | 8.1 | 9.9 |
| Anus | C21 | 0.4 | - | - | - | - | 0.1 | 0.2 | 0.5 | 0.3 | - | 0.4 | 0.5 | 0.7 | - | - | - | 4.9 |
| Liver | C22 | 2.6 | 0.2 | - | - | - | 0.3 | 0.2 | 0.5 | 1.2 | 1.2 | 2.1 | 4.0 | 5.2 | 7.1 | 7.7 | 6.1 | 14.8 |
| Gallbladder etc. | C23-24 | 2.7 | - | - | - | - | - | 0.2 | 0.2 | 0.6 | 1.5 | 2.5 | 10.1 | 2.6 | 5.5 | 10.3 | 10.1 | 9.9 |
| Pancreas | C25 | 1.5 | - |  | - | - | - | 0.2 | 0.2 | - | 0.9 | 1.1 | 2.5 | 4.6 | 4.7 | 2.6 | 4.0 | 14.8 |
| Nose, sinus, etc. | C30-31 | 0.0 | - | - | - | - | - | - | - | - | - | - | - | - | - | 1.3 | - | - |
| Larynx | C32 | 0.1 | - | - | - | - | - | - | - | 0.3 | - | - | - | - | 0.8 | - | - | - |
| Trachea, bronchus, lung | C33-34 | 0.9 | - | - | - | - | 0.1 | 0.2 | - | 0.3 | 0.3 | 1.4 | 0.5 | 1.3 | 3.9 | 3.9 | 2.0 | 4.9 |
| Other thoracic organs | C37-38 | 0.2 | - | - | - | - | 0.1 | - | 0.2 | - | 0.3 | 0.4 | - | 0.7 | - | - | - | - |
| Bone | C40-41 | 2.1 | 0.1 | 0.2 | 0.4 | 1.0 | 0.9 | 1.0 | - | 0.3 | 0.3 | 1.4 | 2.5 | 3.3 | 0.8 | 3.9 | - | - |
| Melanoma of Skin | C43 | 0.4 | - | - | - | - | 0.1 | 0.2 | - | 0.3 | 0.6 | - | 1.5 | - | 0.8 | - | - | - |
| Other skin | C44 | 3.8 | - | - | - | 0.1 | 0.3 | 0.3 | 0.7 | 0.6 | 0.6 | 4.3 | 3.5 | 3.3 | 8.6 | 16.7 | 28.3 | 39.4 |
| Mesothelioma | C45 | 0.0 | - | - | - | - | - | - | - | - | 0.3 | - | - | - | - | - | - | - |
| Connective and soft tissue | C47, C49 | 1.3 | 0.1 | - | 0.2 | 0.3 | 0.7 | 0.3 | 1.4 | 0.3 | 0.9 | 0.4 | 0.5 | 0.7 | 2.4 | 2.6 | - | - |
| Breast | C50 | 27.2 | - | - | - | - | 1.3 | 5.5 | 10.2 | 24.5 | 28.2 | 39.3 | 45.9 | 41.6 | 43.2 | 30.8 | 38.4 | 41.9 |
| Vulva | C51 | 0.5 | - | - | - | - | - | - | - | 0.9 | 0.3 | - | 1.0 | 1.3 | 0.8 | 1.3 | 2.0 | - |
| Vagina | C52 | 0.4 | - | - | - | - | - | 0.2 | 0.2 | 0.3 | - | - | 0.5 | - | 2.4 | 2.6 | - | - |
| Cervix uteri | C53 | 1.9 | - | - | - | - | 0.1 | 0.2 | - | 1.2 | 1.2 | 3.2 | 4.0 | 3.9 | 3.1 | 6.4 | 2.0 | 4.9 |
| Corpus uteri | C54 | 0.9 | - | - | - | - | 0.1 | - | - | 0.6 | 0.6 | 1.1 | 2.0 | 1.3 | 3.1 | 5.1 | - | - |
| Uterus, unspecified | C55 | 1.9 | - | - | - | - | 0.3 | 0.5 | 0.2 | 0.9 | 0.6 | 2.1 | 4.0 | 3.9 | 3.9 | 6.4 | 6.1 | - |
| Ovary | C56 | 3.2 | - | 0.1 | - | 0.5 | 0.4 | 1.0 | 1.2 | 2.6 | 0.9 | 3.9 | 5.0 | 2.6 | 8.6 | 6.4 | 4.0 | 2.5 |
| Other female genital org. | C57 | 1.6 | - | - | - | 0.1 | 0.3 | 1.0 | 0.7 | 1.4 | 0.9 | 1.4 | 3.0 | 2.0 | 3.9 | 1.3 | - | - |
| Placenta | C58 | 0.4 | - | - | - | 0.1 | - | 0.2 | 0.7 | 0.9 | 0.3 | - | - | - | - | - | - | - |
| Kidney | C64 | 2.5 | 1.1 | 0.5 | - | - | 0.3 | 0.3 | 0.2 | 0.3 | 1.2 | 2.9 | 5.0 | 2.6 | 2.4 | 6.4 | 6.1 | 2.5 |
| Renal Pelvis | C65 | 0.0 | - | - | - | - | - | 0.2 | - | - | - | - | - | - | - | - | - | - |
| Ureter | C66 | 0.0 | - | - | - | - | - | - | - | - | - | - | - | - | - | - | - | - |
| bladder | C67 | 0.7 | - | - | - | - | - | - | - | - | 0.3 | 0.4 | 2.0 | 1.3 | 2.4 | 2.6 | 6.1 | - |
| Other urinary organs | C68 | 0.0 | - | - | - | - | - | - | - | - | - | - | - | - | - | - | - | - |
| Eye | C69 | 0.3 | 0.3 | - | - | - | - | - | - | - | 0.3 | 0.4 | - | - | 0.8 | - | - | - |
| Brain, nervous system | C70-72 | 3.4 | 0.3 | 0.6 | 0.5 | 0.5 | 0.7 | 0.5 | 0.7 | 2.9 | 2.1 | 1.4 | 2.5 | 6.5 | 5.5 | 5.1 | 6.1 | 4.9 |
| Thyroid | C73 | 1.4 | - | - | - | - | 0.8 | 0.8 | 0.5 | 1.2 | 0.9 | 1.1 | 1.5 | 1.3 | 1.6 | 3.9 | - | 2.5 |
| Adrenal | C74 | 0.4 | 0.1 | - | - | - | - | 0.3 | - | - | 0.3 | 0.7 | 0.5 | - | 0.8 | 1.3 | - | - |
| Other endocrine | C75 | 0.0 | - | - | - | - | - | - | - | - | - | - | - | - | - | - | - | - |
| Hodgkin lymphoma | C81 | 0.9 | - | 0.3 | 0.3 | 0.3 | 0.3 | 0.2 | 0.7 | 0.3 | - | 0.4 | - | - | 0.8 | 2.6 | 2.0 | 2.5 |
| Non-Hodgkin lymphoma | C82-85,C96 | 2.2 | 0.1 | 0.3 | 0.3 | 0.5 | 0.4 | 1.0 | 0.5 | 0.6 | 0.3 | 2.1 | 2.0 | 1.3 | 1.6 | 9.0 | 4.0 | 2.9 |
| Multiple myeloma | C90 | 0.1 | - | - | - | - | - | - | - | - | - | - | 0.5 | - | - | - | 2.0 | - |
| Lymphoid leukemia | C91 | 1.9 | 0.9 | 1.3 | 1.1 | 0.5 | - | 0.2 | 0.5 | - | - | - | - | 2.0 | - | 1.3 | - | - |
| Myeloid leukemia | C92-94 | 1.4 | 0.5 | 0.6 | 0.6 | 0.4 | 0.3 | 0.8 | - | 0.9 | - | - | - | 1.3 | - | - | - | - |
| Leukemia, unspecified | C95 | 0.9 | 0.8 | 0.2 | 0.6 | 0.1 | - | 0.3 | - | - | - | - | - | 0.7 | 0.8 | - | - | - |
| Other and unspecified | O&U | 10.8 | 0.3 | 0.4 | 0.4 | 0.6 | 1.8 | 2.3 | 2.8 | 2.6 | 7.7 | 8.9 | 15.1 | 8.2 | 27.5 | 20.6 | 28.3 | 39.4 |
| All sites | All |  | 4.9 | 4.4 | 4.2 | 5.3 | 10.5 | 20.9 | 26.0 | 51.4 | 66.0 | 102.3 | 155.8 | 161.2 | 204.3 | 253.2 | 254.8 | 335.3 |
| All sites but C44 | AllbC44 | 100.0 | 4.9 | 4.4 | 4.2 | 5.2 | 10.2 | 20.6 | 25.3 | 50.8 | 65.4 | 98.0 | 152.3 | 158.0 | 195.7 | 236.5 | 226.5 | 295.8 |

| **Table S4.** Comparison of the three most common cancers (by number of cases) among males and females in Kabul with Afghanistan, registries in neighboring countries, Asian regions, Asia, and global estimates. | | | | | | |
| --- | --- | --- | --- | --- | --- | --- |
| ___Regions__ | ____________ Males______________ | | | ____________ Females______________ | | |
|  | 1 | 2 | 3 | 1 | 2 | 3 |
| Kabul | Stomach | Esophagus | Colorectum | Breast | Esophagus | Stomach |
| Afghanistan† | Stomach | Lung | Oral cavity | Breast | Cervix uteri | Stomach |
| Sistan | Stomach | Colorectum | Other skin | Breast | Thyroid | Colorectum |
| Razavi | Stomach | Colorectum | Other skin | Breast | Colorectum | Stomach |
| KPK | Oral cavity | Prostate | Colorectum | Breast | Ovary | Esophagus |
| Lahore | Prostate | Bladder | Lung | Breast | Uterus | Ovary |
| Kathmandu | Lung | Stomach | Oral cavity | Breast | Lung | Cervix uteri |
| Delhi | Lung | Oral cavity | Prostate | Breast | Cervix uteri | Gallbladder |
| South/Central† | Oral cavity | Lung | Esophagus | Breast | Cervix uteri | Ovary |
| West Asia† | Lung | Prostate | Colorectum | Breast | Thyroid | Colorectum |
| Asia† | Lung | Colorectum | Stomach | Breast | Lung | Thyroid |
| Globally† | Lung | Prostate | Colorectum | Breast | Lung | Colorectum |
| Sistan: Sistan & Baluchestan; Razavi: Razavi Khorasan; KPK: Khyber Pakhtunkhwa.  † Based on Globocan 2022 (version 1.1) - 08.02.2024. | | | | | | |
